# Mutational processes in cancer preferentially affect binding of particular transcription factors

**DOI:** 10.1038/s41598-021-82910-0

**Published:** 2021-02-08

**Authors:** Mo Liu, Arnoud Boot, Alvin W. T. Ng, Raluca Gordân, Steven G. Rozen

**Affiliations:** 1 Programme in Cancer and Stem Cell Biology, Duke-NUS Medical School, Singapore, Singapore; 2 Centre for Computational Biology, Duke-NUS Medical School, Singapore, Singapore; 3 Department of Biostatistics and Bioinformatics, Center for Genomic and Computational Biology, Duke University, Durham, NC USA

**Keywords:** Cancer, Computational biology and bioinformatics

## Abstract

Protein binding microarrays provide comprehensive information about the DNA binding specificities of transcription factors (TFs), and can be used to quantitatively predict the effects of DNA sequence variation on TF binding. There has also been substantial progress in dissecting the patterns of mutations, i.e., the "mutational signatures", generated by different mutational processes. By combining these two layers of information we can investigate whether certain mutational processes tend to preferentially affect binding of particular classes of TFs. Such preferential alterations of binding might predispose to particular oncogenic pathways. We developed and implemented a method, termed "Signature-QBiC", that integrates protein binding microarray data with the signatures of mutational processes, with the aim of predicting which TFs’ binding profiles are preferentially perturbed by particular mutational processes. We used Signature-QBiC to predict the effects of 47 signatures of mutational processes on 582 human TFs. Pathway analysis showed that binding of TFs involved in NOTCH1 signaling is strongly affected by the signatures of several mutational processes, including exposure to ultraviolet radiation. Additionally, toll-like-receptor signaling pathways are also vulnerable to disruption by this exposure. This study provides a novel overview of the effects of mutational processes on TF binding and the potential of these processes to activate oncogenic pathways through mutating TF binding sites.

## Introduction

While the oncogenic effects of mutations in the coding sequences of genes have been intensively studied, relatively little is known about the possible oncogenic effects of mutations in noncoding sequence—98% of the human genome. Among noncoding sequences, promoters and other cis-regulatory elements are known to be functionally important, and therefore mutations within these regions are especially likely to contribute to oncogenesis. Transcription factors (TFs) recognize and bind to short DNA sequences (usually ~ 10 nucleotides long), often in the proximal promoter regions of a gene the TF regulates. The binding of the TF then enhances or represses the gene's transcription. A mutation in a TF binding site can lead to a dramatic increase or decrease of TF's binding affinity, and hence the expression of target genes. Despite the potential impact of mutations in promoter regions on oncogenesis, however, at present only a few oncogenic mutations in cis-regulatory regions have been identified. Oncogenic mutation-induced gain of binding sites for ETS (E-twenty-six)-family transcription factors in the promoter of the *TERT* gene is the most prominent example^1,2^.

Protein-binding microarray (PBM) studies have provided systematic, high-throughput data on the effects of sequence changes on TF binding affinity^3,4^. A PBM is a microarray in which each of ~ 40,000 features contains a collection of 60-base-pair duplex DNA probes, all with a particular sequence. A glutathione S transferase (GST)-epitope-tagged TF is allowed to bind to the DNA duplexes on the array, after which a fluorescently labelled anti-GST antibody provides a readout of TF concentration at each feature. A universal PBM is one in which the probes contain multiple instances of all possible 8-mers^5^.

There are several approaches for inferring sequence-dependent changes in binding affinity from universal PBM image intensity data. Here, we use QBiC (Quantitative predictions of TF Binding Changes due to sequence variants)^6^. Briefly, QBiC estimates binding of one TF to a given 6-mer using ordinary-least-squares regression to fit a model in which the log of the fluorescent intensity of a feature is the dependent variable and the number of instances of the given 6-mer in the feature is the independent variable. The fitted coefficient then provides an estimate of the binding. So, for example, when intensity is low despite multiple copies of the 6-mer in the probe sequence, the fitted coefficient will be small, indicating weak binding. Conversely, if intensity rises markedly as a function of the number of 6-mers, the coefficient will be larger, indicating stronger binding. Six different 6-mers overlap a single position, spanning a total of 11 base pairs. Based on the binding change effect of the 6-mers overlapping a single point mutation, QBiC estimates the effect of every single-nucleotide change in the center of every 11-mer. We selected QBiC rather than other approaches to predict binding because of reports that QBiC outperformed DeepBind and position weight matrix (PWM) models in predicting in vitro TF binding changes and allele-specific binding in vivo^6^. The PBM data on which QBiC is based contain information on 582 human TFs.

In parallel with the recent growth of PBM-based TF-binding data, next-generation sequencing has enabled the systematic study of the signatures of mutational processes in 10 s of thousands of tumors^7^. Different mutational processes generate characteristic patterns of mutations in particular sequence contexts, and these patterns can be detected in the somatic mutations in a tumor. For example, skin-cancer genomes bear the signatures of ultraviolet-induced mutations, and most lung cancers bear the signature of mutations caused by tobacco smoking. Henceforth, for brevity, we will refer to the signature of a mutational process as a “mutational signature”. A recent compendium of single-base-substitution mutational signatures comprises 47 non-artifactual, non-clustered, signatures (COSMICv3) extracted from 4645 whole genome and 19,184 exome sequences across most types of cancer^7^ (Catalogue Of Somatic Mutations In Cancer [COSMIC]).

There have been some studies on how particular mutational processes might generate mutations that affect particular genes or pathways. In particular, APOBEC mutagenesis is probably responsible for recurrent mutations in the promoters of the *TBC1D12* and *PLEKHS1* genes in breast cancers^8^. However, we are aware only of a study by Chan et al. that systematically predicted the effects of the signatures of mutational processes on binding of 100 s of TFs^9^. This study assessed the probability that a TFs’ binding affinity would be perturbed by the signature of a particular mutational process. A key output of this analysis was a matrix of the signatures of mutational processes × PWMs, in which each cell contained the probability that a mutation from a given process would cause a gain or loss of binding of the TF recognizing that PWM. The main differences from the study reported here are as follows (1) Chan et al., used Regulatory Sequence Analysis Tool (RSAT^10^) on PWMs to generate a binary classification of mutations as either creating or abrogating binding of TF. In the current study we use PBM data analyzed by QBiC to quantitatively predict increases or decreases in binding affinity. (2) Chan et al., analyzed binding changes across the entire genome, whereas in the current study we focus on proximal promoter regions, which are highly enriched for functional binding sites. (3) Chan et al. analyzed 512 target PWMs over all vertebrates. The current analysis was based on PBM data from 667 experiments that provided information for 582 human TFs. In addition, the current study examined the role of affected TFs in oncogenic pathways, a topic not addressed in Chan et al.

In this study, we developed the "Signature-QBiC" model that integrates signature profiles of mutational processes with the QBiC estimates of changes in binding affinity to investigate the effect of mutational signatures on the binding of 582 human TFs. We then identified biological pathways enriched for TFs with binding that is likely to be affected by the signature of each process.

## Methods

### Mutational signatures and mutation data

**Figure 1:**
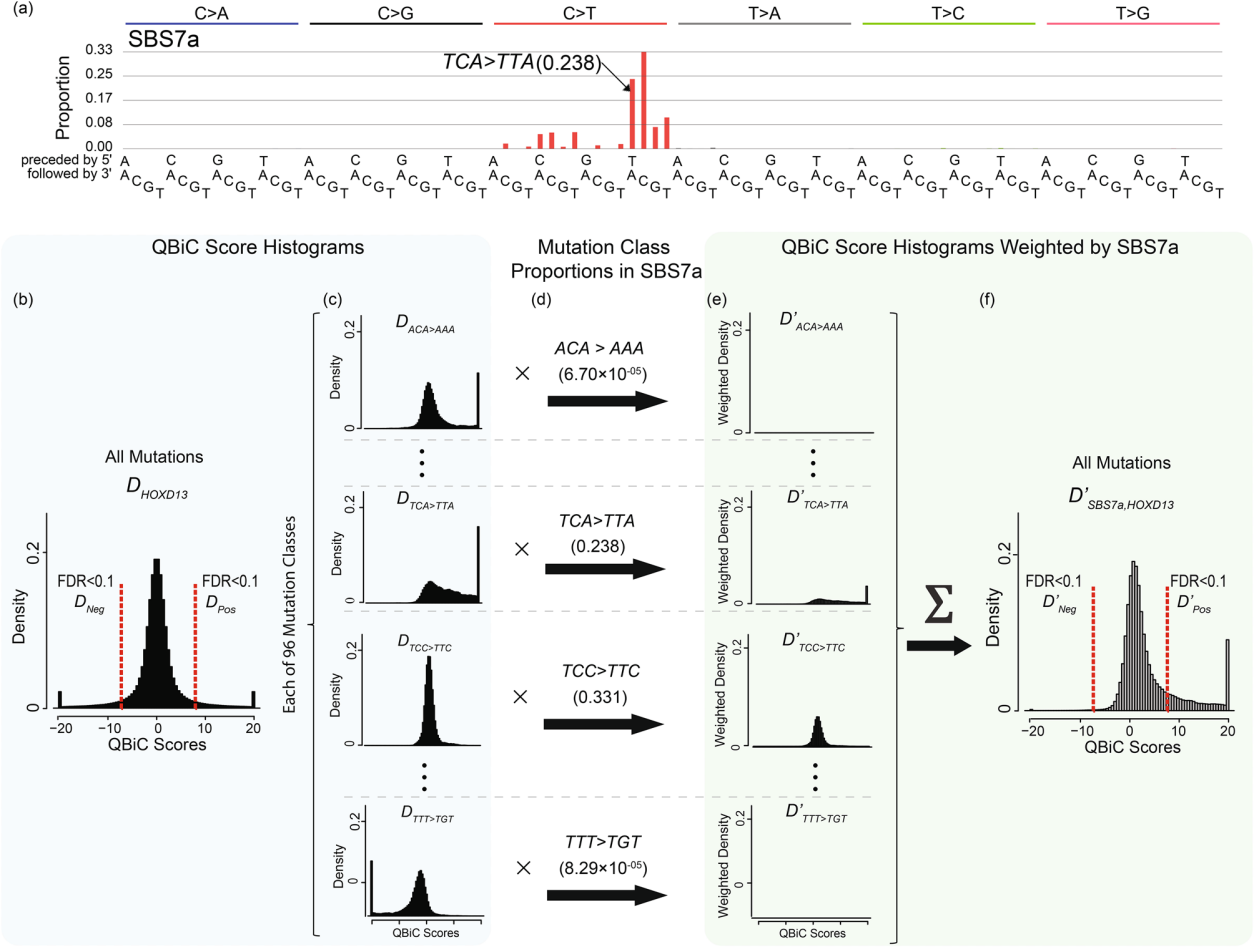
Predicting the effect of mutations due to UV exposure, which generates mutational signature SBS7a, on HOXD13 binding affinity. (**a**) Visualization of mutational signature SBS7a. Central mutations (e.g. C>A) are listed along the top of the xaxis, and source trinucleotides for 96 mutation classes are indicated along the bottom. The height of each bar represents the proportion of mutations due to a particular mutation class. For example, the proportion of TCA>TTA mutations (indicated) is 0.238. (**b**–**f**) Signature-QBiC analysis. (**b**) D_*HOXD*13_ is the histogram of HOXD13 QBiC scores for all 6,291,456 possible 11-mer changes over all possible central trinucleotides. Because there are long tails of extreme QBiC scores, we display the QBiC scores with absolute value ≥ 20 as single bars at the extreme tails of the histogram. (**c**) D_*HOXD*13_ can be decomposed into histograms for each of the 96 mutation classes. Only 4 example mutation classes out of the 96 classes are shown: ACA>AAA (*D*_*ACA*>*AAA*_), TCA>TTA (*D*_*TCA*>*TTA*_), TCC>TTC (*D*_*TCC*>*TTC*_), and TTT>TGT (*D*_*TTT*>*TGT*_). (**d**) We multiply each histogram in (**c**) by the proportions of the corresponding mutation classes in mutational signature SBS7a, to get (**e**) 96 histograms weighted by the expected frequencies of mutations due to SBS7a: $D_{ACA > AAA}^{\prime} , \ldots ,D_{TCA > TTA}^{\prime} ,D_{TCC > TTC}^{\prime} , \ldots D_{TTT > TGT}^{\prime}$. (**f**) The sum of all 96 weighted histograms in (**e**) yields $D_{SBS7a,HOXD13}^{\prime}$, which is the histogram of HOXD13 QBiC scores for all mutations weighted by the expected frequencies of mutations due to SBS7a. In (**a**) and (**f**), red dashed lines at QBiC scores corresponding to FDR < 0.1 demarcate the *D*_*Pos*_, *D*_*Neg*_, $D_{Pos}^{\prime}$, and $D_{Neg}^{\prime}$ portions of the histograms.

We adopt common usage, in which we analyze the signatures of mutational processes in terms of the relative proportions of mutations in each of the mutation classes ACA>AAA, ACA>AGA, ACA>ATA, CCA>CAA, …, TTT>TGT^11^. These mutation classes consist of all single nucleotide substitutions in the context of the immediately preceding and following bases. These mutation classes do not take into consideration the DNA strand of the central mutated base, and by convention we reverse complement the source trinucleotide if the mutated base is a purine, i.e. A or G. For example, AGC>ATC mutations are grouped with GCT>GAT mutations. Thus, there are 96 mutation classes: 4 bases 5′ of the mutated base × 2 possible mutated bases [C or T] × 3 possible central bases after the mutation × 4 bases 3′ of the mutated base. We can view the signature of a mutational process as a multinomial distribution that describes the probability that a new mutation will be one of the possible 96 classes. Figure 1a shows an example of signature SBS7a, which is caused by ultraviolet radiation. The height of each vertical bar indicates the proportion of mutations in a particular mutation class. For example, in SBS7a, mutations from TCC>TTC, with a probability of 0.331, are the most common, and other mutations from TCN>TTN are also common (the red bars near the middle of the plot). We use *σ* to denote a mutational signature and subscript its elements by mutation class. For example, in SBS7a, $\sigma_{TCA > TTA} = 0.238$ (Fig. 1a). We used the non-artifactual single-base-substitution mutational signatures from COSMIC Mutational Signatures v3.0 (https://cancer.sanger.ac.uk/cosmic/signatures/SBS/)^7^.

We downloaded signature exposure data from https://www.synapse.org/#!Synapse:syn11738669 and mutation calls from https://dcc.icgc.org/api/v1/download?fn=/PCAWG/mutational_signatures/Input_Data_PCAWG7_23K_Spectra_DB/vcf_like_simple_files/WGS_Other.20180413.simple.gz^7^.

### Estimating the effect of mutations on binding change

As described above, for a given TF, for each single-nucleotide change in the center of an 11-mer, QBiC provides a score that estimates the effect of that change on the binding of the TF. For example, for the TF HOXD13 (Homeobox D13), the QBiC-score for AAAATCCGGAA>AAAATTCGGAA is 22.61 (ranking in the 99th percentile of all QBiC-scores), with a QBiC estimated *p* = 3.024 × 10^−113^. The low *p* value and high QBiC score indicate high confidence that this mutation strongly increases binding with HOXD13. QBiC-scores can be positive or negative: positive QBiC-scores indicate increased TF binding affinity and negative scores indicate reduced TF’s binding affinity. For this study, we used the "prediction" (i.e. the QBiC score) and "*p* value" tables downloaded from http://qbic.genome.duke.edu/download/ on June 4, 2019. QBiC-Pred used 667 high-quality PBM experiments which tested human TFs as well as homologous TFs with high amino-acid identity in the DNA-binding domain region (more details in^6^). These PBM experiments were mapped to 582 human TFs. The binding domain classifications for these TFs were downloaded from http://humantfs.ccbr.utoronto.ca/download/v_1.01/DatabaseExtract_v_1.01.csv on June 10, 2019 (reference^12^).

### Integrating mutational signatures with QBiC scores

For a given signature and TF, we can combine the probability of a mutation of a given mutation class (e.g. TCA>TTA) with the QBiC predictions of binding changes (QBiC scores and *p* values) in all 11-mers centered on that mutation (e.g. centered on TCA). There are 4^(11−3)^ = 65,536 11-mers centered on TCA, each of which can undergo 3 mutations at the central nucleotide (C>A, C>G, C>T). Thus, there are a total of 196,608 possible 11-mer changes for a given central trinucleotide, and 6,291,456 for changes from the 32 possible central trinucleotides centered on C or T. Assuming that mutations in all mutation classes occur with equal frequency, we can plot the distribution of the QBiC scores of these 6,291,456 changes as a histogram that (e.g. *D*_*HOXD13*_ in Fig. 1b). Then, for a given signature (e.g. SBS7a), we can multiply the probability of mutations in each mutation class (e.g. TCA>TTA) in the signature times the distribution of QBiC scores of the 65,536 11-mer changes associated with that mutation class (Fig. 1c–e). This results in a new histogram ($D_{TCA > TTA}^{\prime}$ in Fig. 1e). Then, to get $D_{\sigma }^{\prime }$, the expected distribution of QBiC scores for a given TF due to mutations induced by a given signature (e.g. $D_{SBS7a,HOXD13}^{\prime}$ in Fig. 1f, where SBS7a is *σ* and the subscript HOXD13 indicates the TF in question), we compute

1 $$D_{\sigma }^{\prime} = \mathop \sum \limits_{{\mu \in {\rm M}}} \sigma_{\mu } \cdot D_{\mu } ,$$

where *μ* is one mutation class, *M* is the set of 96 mutation classes [ACA>AAA, ACA>AGA, …, TTT>TGT], *σ*_*μ*_ is the probability of *μ* in signature *σ*, and *D*_*μ*_ is the distribution of QBiC scores for a given TF and a given mutation class *μ* (e.g. $D_{TCA > TTA}$) assuming equal frequency of all mutation classes. Figure 1b–f shows the computation of $D_{SBS7a}^{\prime}$ for the example of the TF HOXD13.

We will define Gain Ratio (GR) and Loss Ratio (LR) to indicate whether a TF’s binding affinity likely increases or decreases as a result of the expected distribution of mutation types generated by a particular mutational process. GR and LR are computed from the comparison between (1) the expected distribution of QBiC scores based on the assumption that all mutations occur with equal frequency versus (2) the expected distribution of scores based on the assumption that the frequency of different mutations depends on the signature of the mutational process. As noted above, for each TF, QBiC provides scores for 6,291,456 mutations. For each TF, it also provides a *p* value for each of the 6,291,456 mutations. This is the *p* value for the null hypothesis that the mutation does not change PBM intensity (which is a proxy for binding) versus the alternate hypothesis of a change in PBM intensity. For a given TF, let *D*_*Pos*_ be the expected distribution of positive QBiC scores that have a Benjamini–Hochberg FDR < 0.1 under the assumption that all mutations occur with equal frequency (Fig. 1b). The *p* value is a strictly decreasing function of the absolute value of the QBiC score. Therefore, *D*_*Pos*_ is also the distribution of QBiC scores > a particular QBiC score, *T*. We define *D*_*Neg*_ analogously for mutations with negative QBiC scores. For a given signature, *σ*, we then define $D_{Pos}^{\prime }$, as the portion of the distribution $D_{\sigma }^{\prime }$ (Eq. 1) with QBiC scores > *T* (equivalent to Benjamini–Hochberg FDR < 0.1, Fig. 1f). We note that since *T* is the same for both *D*_*Pos*_ and $D_{Pos}^{\prime }$, both distributions comprise the same mutations; only the expected frequencies of the mutations change between *D*_*Pos*_ and $D_{Pos}^{\prime }$. We define $D_{Neg}^{\prime}$, analogously.

Finally, we want to know if, given the mutations caused by the mutational process that generates the signature, there are more mutations that increase binding than under the assumption that all mutations arise with equal frequency, i.e. if $area\left( {D_{Pos}^{\prime} } \right) > area\left( {D_{Pos} } \right)$. We assessed whether $area\left( {D_{Pos}^{\prime} } \right)$ is statistically > than *area*(*D*_*Pos*_) by testing the null hypothesis that $area\left( {D_{Pos}^{\prime} } \right) - area\left( {D_{Pos} } \right)$ is no greater than expected by chance given random mutations assuming that all mutations arise with equal frequency (function ResampleMutationFrequency in our code file at https://github.com/liumoLM/SigQBiC/blob/master/Code_for_paper/ExampleOfSignatureQBiC.pdf). We tested whether $area\left( {D_{Neg}^{\prime} } \right) - area\left( {D_{Neg} } \right)$ is statistically significant using the same procedures. We also define

2 $$GR = { }area\left( {D^{\prime}_{Pos} } \right)/area\left( {D_{Pos} } \right)$$

and

3 $$LR = { }area\left( {D^{\prime}_{Neg} } \right)/area\left( {D_{Neg} } \right)$$

If a TF was assayed in multiple PBM experiments, we used the median GR and LR from those experiments.

### Genome annotation

We identified the locations of genes and transcription start sites from human genome reference sequence GRCh37 (http://hgdownload.cse.ucsc.edu/goldenPath/hg19/bigZips/hg19.fa.gz) and GENCODE release 27 (ftp://ftp.ebi.ac.uk/pub/databases/gencode/Gencode_human/release_27 /GRCh37_mapping/gencode.v27lift37.annotation.gtf.gz).

### Code availability

Signature-QBiC and related code are available from https://github.com/liumoLM/SigQBiC.

## Results

### TF binding-change predictions based on signatures alone are consistent with predictions based on actual mutations in tumors

Mutation rates and signature profiles in proximal promoter regions might be altered by local characteristics, such as binding by protein complexes involved in transcription and transcriptional regulation or DNA damage and repair stemming from transcriptional initiation^13^. Therefore, we compared TF binding-change predictions based on mutational signatures alone (e.g. Fig. 1f) with TF-binding-change predictions based on observed proximal promoter mutations in actual tumors. For this analysis, we selected signatures that have identified etiologies and, importantly, often dominate the mutational spectra of affected tumors: SBS2, SBS4, SBS7a, SBS10a, SBS13, SBS17b, and SBS22 (Table 1). We analyzed mutations in the proximal promoter regions of tumors in which ≥ 40% of mutations across the whole genome were due to one of these mutational signatures^7^.

**Table 1:** Summary of tumors dominated by particular mutational signatures, selected for analysis of mutational signatures in proximal promoters.

Signature	Etiology	Cancer types	Major mutation classes	Number of tumors	Total number of mutations in proximal promoters^a^
SBS2	Activated APOBEC	Breast, pancreatic, etc	C>T	9	9,339
SBS4	Tobacco smoke	Lung and head & neck	All	45	117,413
SBS7a	UV radiation	Skin melanoma	C>T	80	413,834
SBS10a	Defective polymerase epsilon proofreading	Colorectal and uterine	C>A	6	86,917
SBS13	Activated APOBEC	Breast, pancreatic, etc	C>A, C>G	16	13,895
SBS17b	Unknown	Esophageal and stomach	T>G	11	8,750
SBS22	Aristolochic acids	Liver and kidney	T>A	6	3,709

Table S1 provides details.

^a^Regions from 2000 bp 5′ to 2000 bp 3′ of transcription start sites.

**Figure 2:**
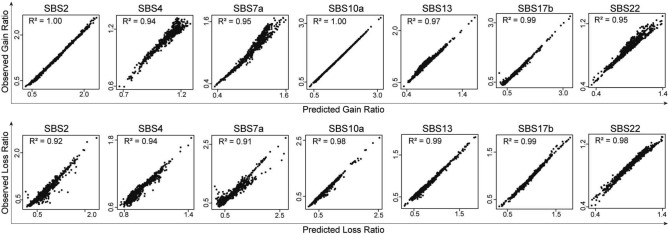
Gain and loss ratios based on the frequencies of mutations in the signatures were similar to gain and loss ratios based on actual promoter mutations observed in tumors with mutations dominated by a particular signature. Each dot corresponds to a PBM experiment for one TF.

We generated the aggregate proximal-promoter mutation spectrum observed for each signature (Table 1), and for each signature's aggregate spectrum we calculated the GR and LR for each PBM experiment. To minimize interference from other signatures, for all signatures except SBS4, we only analyzed mutations in the major mutation classes present in the signature. For each signature, we selected the major mutation class or classes (i.e. mutations of single nucleotides in isolation) that collectively contribute > 90% of the mutational signature. For example, SBS2 consists almost exclusively of C>T mutations (99.24% of the signature). Therefore, we only analyzed the C>T mutations in the heavily SBS2-mutated tumors. GRs and LRs based on the signature profile alone (Fig. 2) were highly correlated with the GRs and LRs based on actual promoter mutations (mean R^2^ 0.965). Thus, atypical characteristics of mutational processes in promoters do not distort the mutational signatures. We therefore based further analyses on GRs and LRs computed from the mutational signature alone according to equations (Eq. 2) and (Eq. 3).

### Comprehensive analysis of TF binding affinity alteration caused by mutational signatures

**Figure 3:**
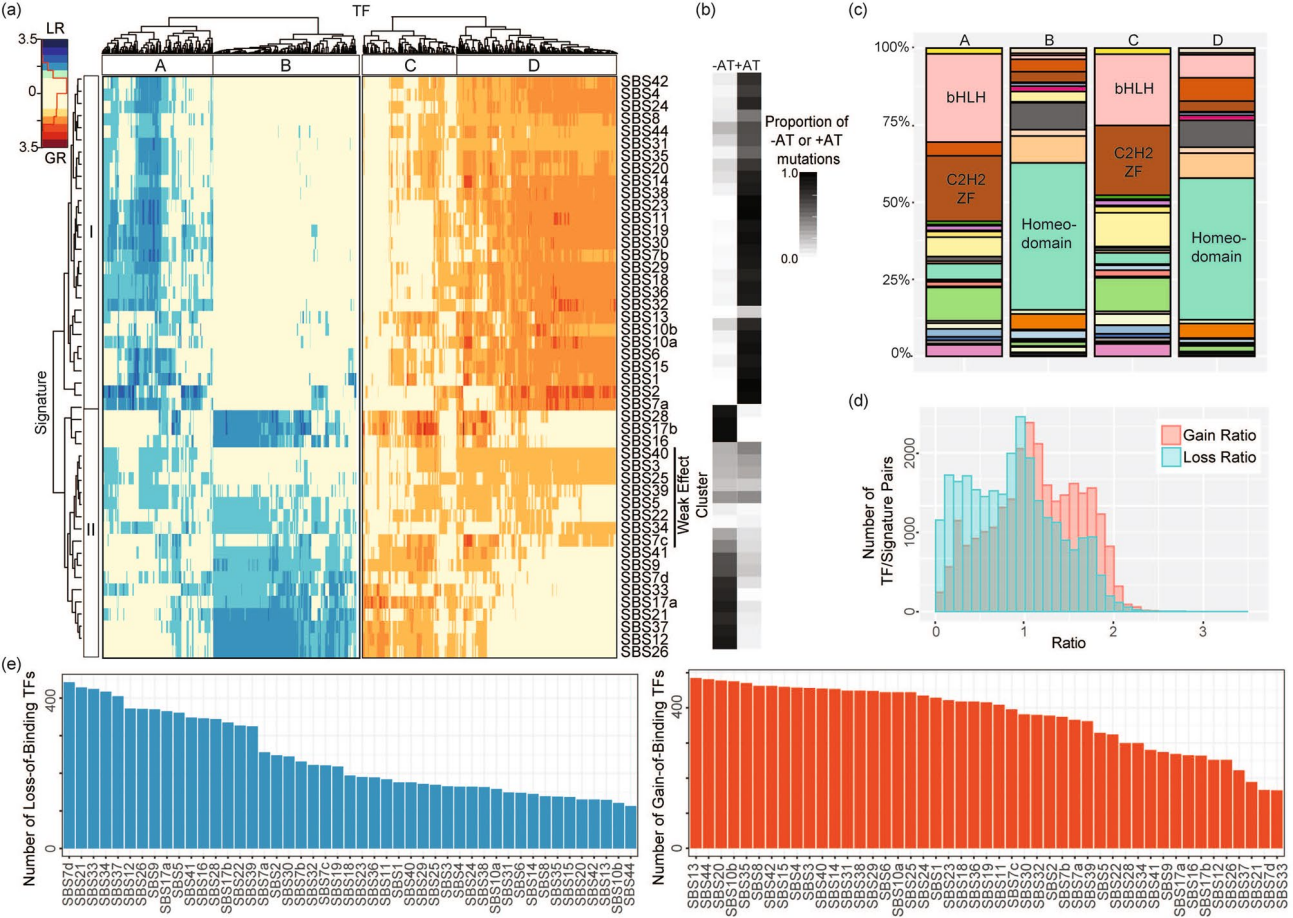
Overview of mutational signatures’ effects on TF binding affinity. (**a**) Heatmap of GRs and LRs for 582 human TFs (columns) and 47 mutational signatures (rows). Both dimensions were grouped by the R hclust function using unsupervised hierarchical clustering with complete linkage on Euclidean distance. (**b**) The proportions of mutations in each signature that mutate cytosine or guanine to adenine or thymine (labelled "+ AT") or vice-versa (labelled "–AT"). Each row corresponds to a signature labelled in (**a**). (**c**) Binding-domain classes within each TF cluster in (**a**). Most frequently affected classes in each cluster are labelled. bHLH, basic helix-loop-helix; C2H2 ZF, C2H2-zinc finger. Additional details in Figure S1 and Table S4. (**d**) Histograms of GRs and LRs for all pairs of TFs and mutational signatures. (**e**) The numbers of gain- and loss-of-binding TFs for each of the 47 mutational signatures.

We used the mutational-signatures and QBiC scores and *p* values to determine the GRs and LRs of 582 human TFs for each of 47 mutational signatures (Tables S2 and S3). When a given TF was represented by > 1 PBM experiments, we used the median of GRs or LRs. Clustering of signatures by GR and LR across the 582 TFs identified 2 major clusters: I and II on the left of Fig. 3a. Clustering of TFs by GR and LR across the 47 mutational signatures identified 4 major groups of TFs (Table S4): A through D along the top of Fig. 3a. Every cluster of TFs was predominantly affected by certain clusters of mutational signatures: TF Cluster A was characterized by high LRs in Signature Cluster I, B by high LRs in Signature Cluster II, C by relatively high GRs in Signature Cluster II, and D by high GRs in Signature Cluster I. As expected, we observed that for a given TF, mutational signatures that cause a gain of binding usually do not result in a loss of binding, and vice versa. This is clearly reflected in the overlap in clusters B and D, where > 80% of the TFs overlap (*p* < 2.2 × 10^−16^, Fisher’s exact test).Some of the TF clusters are dominated by one or a few TF classes, which partly accounts for the pattern of clustering (Fig. 3a, c). TF clusters A and C mainly contain TFs with C2H2 zinc finger (C2H2 ZF) or basic helix-loop-helix (bHLH) binding domains. TF clusters D and B consist largely of TFs with homeodomains, with TFs in Cluster D having high GRs for Cluster I signatures, and TFs in Cluster B having high LRs for Cluster II signatures. For TF Cluster D, signatures in Cluster I are dominated by mutations from C>A or C>T (denoted “+ AT” mutations in Fig. 3b) which results in the creation of the AT-rich homeodomain recognition sequence $$\begin{array}{*{20}c} {5^{\prime} {\text{TAAT }}3^{\prime} } \\ {3^{\prime} {\text{ATTA }}5^{\prime} } \\ \end{array}$$^14^. For example, signature SBS2 consists of 99% C>T mutations, and these cause high GRs in most TFs in Cluster D (Fig. 3a, b). As an example of a TF strongly affected by SBS2, nearly 80% of the large SBS2-associated GR for the PHOX2A (Paired Like Homeobox 2A) homeodomain TF is due to TCA>TTA mutations, which constitute > 50% of SBS2 mutations (Table S5). Thus, SBS2 induces many mutations from $$\begin{array}{*{20}c} {5^{\prime}{\mathbf{T}} {\varvec{G}}{\mathbf{A}}{\text{T }}3^{\prime} } \\ {3^{\prime} {\mathbf{A}}{\varvec{C}}{\mathbf{T}}{\text{A }}5^{\prime} } \\ \end{array}$$ to the PHOX2A recognition sequence $$\begin{array}{*{20}c} {5^{\prime} {\mathbf{T}}{\varvec{A}}{\mathbf{A}}{\text{T }}3^{\prime} } \\ {3^{\prime} {\mathbf{A}}{\varvec{T}}{\mathbf{T}}{\text{A }}5^{\prime} } \\ \end{array}$$ (shown conventionally from the perspective of the bottom strand as TCA>TTA).

In the second case, TFs in Cluster B have large LRs caused by Cluster II signatures which are often dominated by T>C and T>G mutations (denoted “-AT” mutations in Fig. 3b). For example, SBS26, 84% of which consists of T>C mutations, has a high LR in most TFs in Cluster B. This is because T>C mutations disrupt the AT-rich homeodomain recognition sequence.

In addition, within Signature Cluster II, there is a “weak-effect" sub-cluster consisting of SBS3, SBS5, SBS22, SBS25, SBS34, SBS39 and SBS40 (Fig. 3a). Signatures in this sub-cluster do not have strong effects on any TF. This set of signatures can be further subdivided into 2 groups. First, there are the so-called "flat signatures", SBS3, SBS5, SBS25, SBS39 and SBS40, which show mutagenesis in all 6 mutation types. Because of this, they cause both gain and loss of binding mutations, thus they do not affect any TF in a very specific manner, resulting in low GRs and LRs (Figure S2). The remaining signatures in the weak-effect sub-cluster (SBS22 and SBS34) are those that consist mostly of T>A mutations (Figure S2).

In general, mutational signatures had larger GRs than LRs (Fig. 3d, Wilcoxon test *p* < 2.2 × 10^−16^), but this trend varied among TF binding-domain classes (Figure S3). However, a strong difference was observed between signatures. For example, SBS13 had GRs > 1 for 83.3% of TFs, whereas SBS7d had LRs > 1 for 75.9% of TFs; SBS33 had GRs > 1 for 28.4% of TFs and SBS44 has LRs > 1 for 19.4% of TFs (Fig. 3e).

### Relationships between + AT and − AT signatures, change in binding, and change in entropy

**Figure 4:**
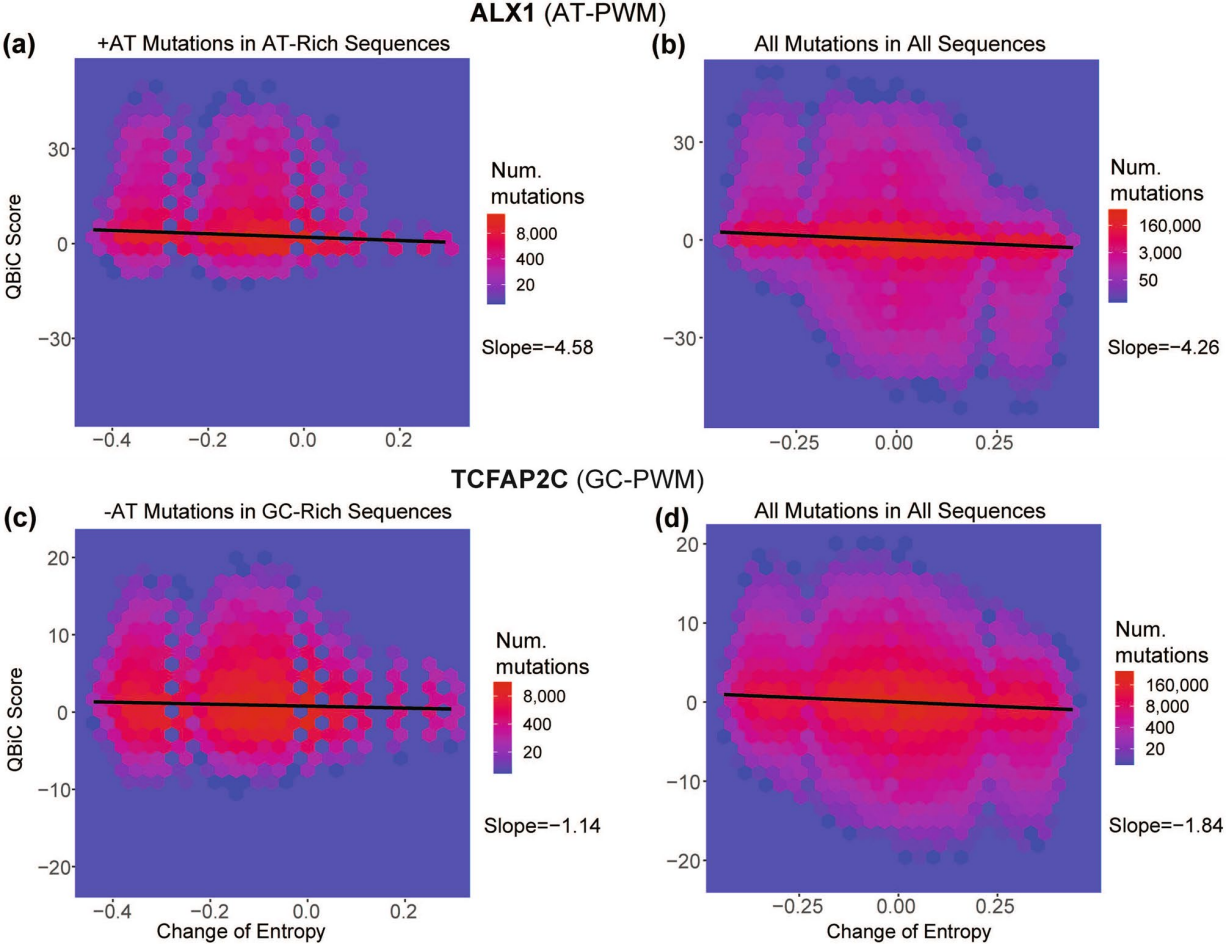
Relationships between change in QBiC-score and change in entropy due to + AT mutations or − AT mutations in TFs with an AT-PWM or a GC-PWM. (**a**) ALX1 has an AT-PWM (72% average AT content in the PWM). For ALX1, + AT mutations in AT-rich sequences tend to decrease entropy (median − 0.11, MAD 0.09) and have positive QBiC-scores (median 1.53, MAD 1.88). Larger decreases in entropy are weakly associated with higher QBiC scores (slopes as shown). (**b**) By contrast, over all mutations in all sequences the median change in entropy and the median QBiC score are 0 (MAD of QBiC score 0.10, MAD of entropy 1.36, one-sided Wilcoxon rank-sum test versus + AT mutations in AT-rich sequences *p* < 2.2 × 10^−16^). Larger decreases in entropy are still weakly associated with higher QBiC scores. (**c**) TCFAP2C has a GC-PWM (32% average AT content in the PWM). For TCFAP2C, − AT mutations in GC-rich sequences tend to decrease entropy (median − 0.11, MAD 0.09) and have positive QBiC scores (median 0.47, MAD 2.28). Larger decreases in entropy are weakly associated with higher QBiC-scores. (**d**) By contrast, over all mutations in all sequences the median change in entropy and the median QBiC score are 0 (MAD of QBiC score 0.10, MAD of entropy 1.50, one-sided Wilcoxon rank-sum test versus − AT mutations in GC-rich sequences *p* < 2.2 × 10^−16^). Larger decreases in entropy are still weakly associated with higher QBiC scores. Change of entropy calculated by the “entropy” function in the DescTools package (https://CRAN.R-project.org/package=DescTools); slopes computed by the rlm function in the MASS package, https://CRAN.R-project.org/package=MASS). Num. mutations indicates the number of mutations in 11-mer context in each hexagonal bin. AT-rich sequences are > 60% AT; GC-rich sequences are < 40% AT.

We investigated the relationships between (1) signatures dominated by + AT mutations, which tend to increase entropy of AT-rich sequences and (2) increased binding of TFs that recognize motifs with high proportions of AT. We also investigated the converse relationships for signatures dominated by − AT mutations. First, using each TF’s PWM as derived from PBM experiments (http://cisbp.ccbr.utoronto.ca/), we categorized TFs as “AT-PWM TFs” (AT percent > 60%) and “GC-PWM TFs” (AT percent < 40%), where AT percent was the average of the sum of the A and T cells in the PWM. For TFs assayed with multiple PBMs we used the median AT percent. There were 321 AT-PWM TFs and 74 GC-PWM TFs (Table S4) To further analyze the effect of + AT signatures on AT-PWM TFs, for each + AT signature, we examined the expected change in entropy of “AT-rich sequences” (AT-content > 60%) given the expected proportions of different mutation classes in the mutational signature, by multiplying the entropy change for each mutation by the mutation’s frequency in the signature. We found that + AT signature mutations in AT-rich sequences tend to increase binding of AT-PWM TFs (columns 2 and 3 in Table S8). Conversely, − AT signature mutations in “GC-rich sequences” (< 40% AT), also usually increase binding of GC-PWM TFs (columns 2 and 3 in Supplementary Table S9), although the effect is less pronounced. However, for each individual TF, the connection between change in entropy and QBiC score is variable. Figure 4 shows two examples. One is the effect of + AT mutations in AT-rich sequences on binding by ALX1, a TF with an AT-PWM. These mutations tend to decrease entropy (median change − 0.11, median absolute deviation [MAD] 0.09) and increase binding (median QBiC score 1.53, MAD 1.88, Fig. 4a). For all mutations in all sequences the median change in entropy and median QBiC score are 0 (Fig. 4b, MAD for change in entropy 1.36, MAD of QBiC scores 0.10). The converse example is the effect of − AT mutations in GC-rich sequences on binding by TCFAP2C, a TF with a GC-PWM. These mutations also tend to reduce entropy (median change − 0.11, MAD 0.09) and increase binding (median QBiC score 0.47, MAD 2.28, Fig. 4c). For all mutations in all sequences the median change in entropy and median QBiC score are 0 (Fig. 4d, MAD for change in entropy 1.50, MAD of QBiC scores 0.10).

### Pathways enriched for TFs affected by mutational signatures

Given that we can predict how the frequencies of mutation types induced by a particular mutational process with a particular signature are likely to affect the binding of a TF, it is natural to ask whether the set of TFs affected by the process are overrepresented in any pathways. To do this, for a given mutational process and its associated mutational signature, we define the pair as “gain-of-binding” if $area\left( {D_{Pos}^{\prime} } \right) - area\left( {D_{Pos} } \right)$ is significantly > 0. We define the pair as “loss-of-binding” if $area\left( {D_{Neg}^{\prime} } \right) - area\left( {D_{Neg} } \right)$ is significantly > 0 (Table S10 and S11). For each signature, we used the R enrichR::enrichr function to search for enrichment of the gain-of-binding TFs for that signature against the Reactome 2016 database^15,16^. We used the same procedure for loss-of-binding TFs. In total, we identified 90 pathways that are significantly enriched for gain- or loss-of-binding of TFs for at least one signature (q < 0.005, as computed by enrichr using Fisher's exact tests and Benjamini–Hochberg false discovery rates, Figure S4; Tables S6, S7 list the TFs driving enrichment for each pathway).

**Figure 5:**
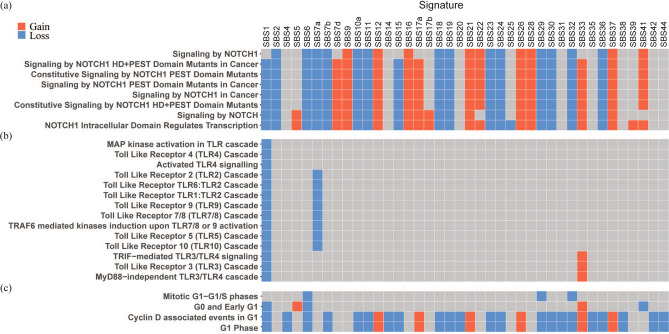
Signatures with pathways enriched for gain or loss-of-binding TFs. (**a**) NOTCH1 pathways. (**b**) TLR (toll-like receptor) signaling pathways. (**c**) G0-G1-S phase pathways.

Among these 90 pathways, it is notable that 8 NOTCH1-related pathways were enriched for TFs with gain or loss of binding due to a large number of signatures (Fig. 5a). NOTCH1 pathways are dysregulated in skin and esophageal cancer which are dominated by SBS7a, SBS7b and SBS17a^17,18^, and thus these signatures may tend to promote this dysregulation. There are also 13 toll-like receptor (TLR) signaling pathways enriched for TFs with gain or loss of binding due to SBS1 and SBS7a (Fig. 5b). Skin melanomas usually have many SBS7a mutations, which are caused by UV radiation, and, consistent with SBS7a mutations affecting TLR promoters, abnormal TLR expression and signaling have been reported in skin melanomas^19,20^. Additionally, 4 G0-G1-S phase pathways were enriched for TFs that are affected by several signatures including SBS1 and SBS6 (Fig. 5c). SBS1 signatures tend to accumulate with age in all cells, and SBS6 is caused by defective DNA mismatch repair^21,22^.

### Comparison with Chan et al.

We are aware of a single previous study in this area, one reported by Chan et al.^9^. This study made binary predictions of gain or loss of TF binding. These predictions were based on the *p* values for mutation-induced sequence changes that were computed by the matrix-scan function in RSAT (Regulatory Sequence Analysis Tool^10^) using PWM representations of recognition sequences. We therefore refer to this as the “RSAT/PWM” method.

**Figure 6:**
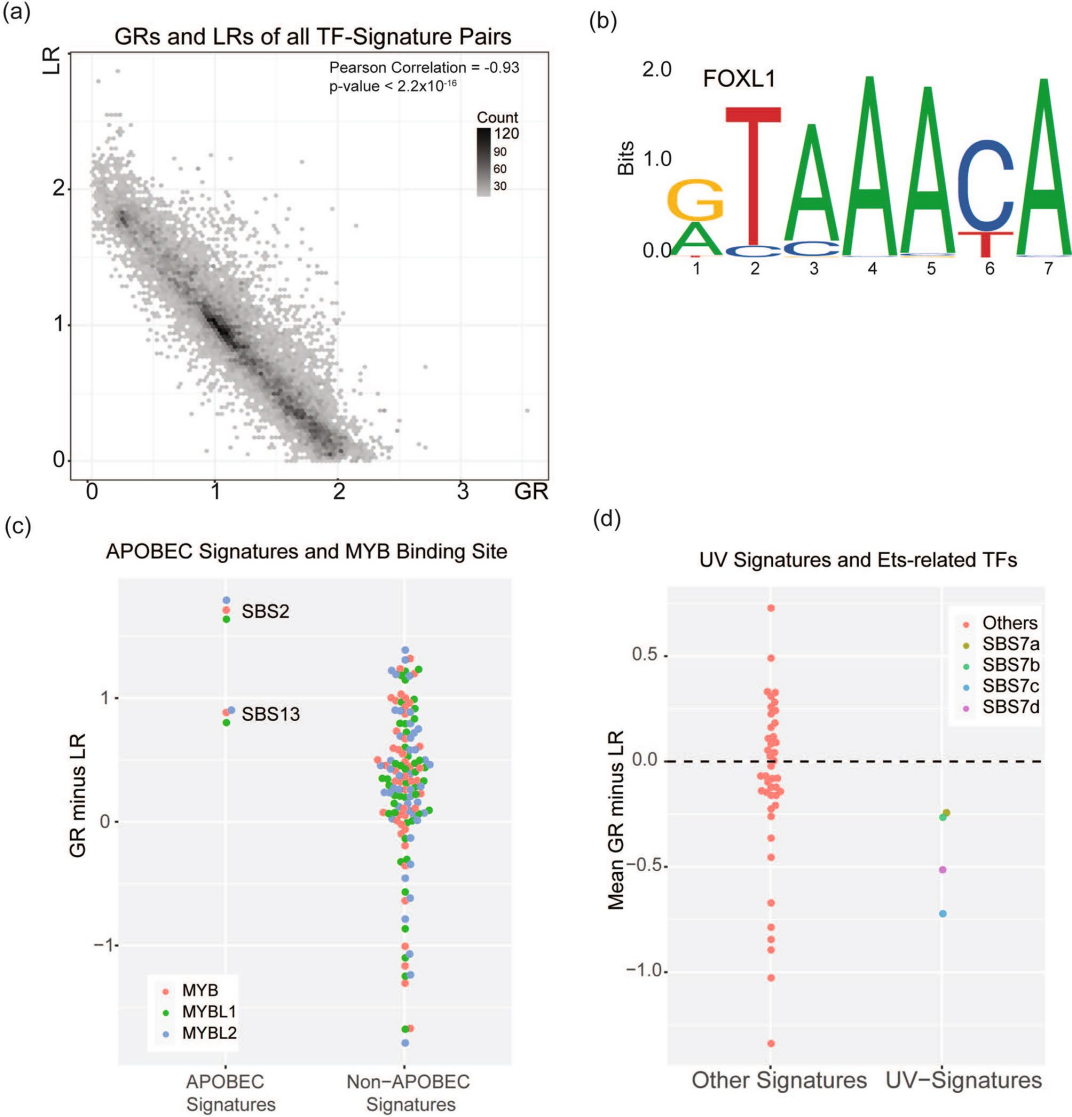
Comparison with the RSAT/PWM approach. (**a**) Unlike RSAT/PWM’s finding of correlation between motif-disruption and motif-creation probabilities, Signature-QBiC GRs and LRs were anti-correlated over all TF-signature pairs. GR, gain ratio; LRs loss ratio. Each dot represents a TF-signature pair. (**b**) FOXL1 PWM logo (downloaded from http://jaspar.genereg.net/static/logos/svg/MA0033.2.svg on December 23, 2019). NGC>NTG (GCN>GAN) mutations generating adenines at positions 3, 4 or 7 enhance FOXL1 binding affinity. However, the effects at positions 3 and 4 are relatively weak, while the effect at position 7 is strong, and RSAT/PWM does not take mutations at this position into consideration. (**c**) Like RSAT/PWM, Signature-QBiC supports the finding that APOBEC signatures tend to increase MYB binding. Like the “alteration offset” in Chan et al., GR minus LR captures the difference between the probabilities of a gain of binding and a loss of binding as the result of the mutational signature caused by a particular mutational process. Three TFs with MYB binding sites (MYB, MYBL1 and MYBL2) represented by PBM experiments M01855_1.94d, M01856_1.94d and M01854_1.94d, respectively. (**d**) Signature-QBiC supports Chan et al.’s conclusion that UV signatures (SBS7a, SBS7b, SBS7c and SBS7d) tend to disrupt binding of ETS-TFs. “Mean GR minus LR”, which is the mean value of GRs minus LRs of 28 TFs, is analogous to “mean differential alteration probability” in Chan et al.’s analysis. This analysis comprises 28 ETS-family TFs represented by 29 PBM experiments (details in Table S4).

Across all TFs and signatures, RSAT/PWM predicted a correlation between motif disruption and motif creation. By contrast, Signature-QBiC found GRs and LRs to be strongly anticorrelated (Fig. 6a, Spearman correlation − 0.93, *p* < 2.2 × 10^−16^). Chan et al. did not publish their code, so we were unable to systematically investigate all the differences that led them to conclude a correlation between disruption and creation. However, this difference may partly stem from the fact that RSAT/PWM did not capture information on mutations at the first and last base pairs of PWMs. For example, Signature-QBiC predicted that signature SBS1 would tend to cause strong gain of binding and weak loss of binding by FOXL1 (GR 1.22, LR 0.18). However, RSAT/PWM’s published prediction was that the chances of creating or destroying a binding site were similar. Examination of the FOXL1 PWM (Fig. 6b) shows that a FOXL1 binding site can be created by NCG>NTG mutations (CGN>CAN on the complementary strand), which make up 89% of SBS1 mutations. In particular, these mutations can generate an adenine at position 7 and thereby enhance FOXL1 binding affinity, but mutations at this position are not considered by RSAT/PWM. While NCG>NTG (CGN>CAN) mutations can also generate new adenines at positions 3 or 4, the preceding cytosines are only weakly favored, as shown in the logo. Therefore, mutations at bases 3 or 4 barely affect FOXL1 binding. Conversely, none of the main mutation types in signature SBS1 disrupt the FOXL1 binding motif. Therefore, the Signature-QBiC prediction of strong gain of binding due to SBS1 mutations seems well supported by the PWM, while RSAT/PWM did not consider the critical mutation at position 7.

Nevertheless, Signature-QBiC concurred with two other results reported by Chan and colleagues. First, like RSAT/PWM, Signature-QBiC confirmed a previous report that APOBEC signatures tend to create rather than destroy MYB binding sites (Wilcoxon rank-sum test, *p* = 3.18 × 10^−9^, Fig. 6c)^23^. RSAT/PWM also predicted that mutations caused by UV radiation (signature SBS7 in COSMICv2^24^,) tend to disrupt binding sites across ETS-family TFs. Signature-QBiC was concordant: it found that signatures caused by UV exposure (SBS7a, SBS7b, SBS7c and SBS7d in COSMICv3 (reference^7^) tend to disrupt binding of ETS-family TFs (Fig. 6d).

## Conclusion and discussion

While several studies have searched for noncoding driver mutations in cancer^25–27^, there has been little systematic study of the overall likely effects of mutational signatures on TF binding. Indeed, we are aware of only one previous study in this area^9^, and we refer to the method in this study as the “RSAT/PWM method”. We believe Signature-QBiC provides two advantages over RSAT/PWM. First, compared to the binary assessments of differences in binding in RSAT/PWM, PBM experiments provide nuanced, quantitative estimates of differences in binding affinity. Second, RSAT/PWM did not consider mutations that change the first or last base pair of the PWM. In addition, RSAT/PWM was applied to the entire genome sequence, rather than proximal promoter regions, which in general are enriched for functionally important TF binding. On the other hand, Signature-QBiC uses universal PBM data, which only captures information on binding to 11 bp sequences, and therefore provides limited information on the binding-sequence preferences of the relatively few TFs with longer recognition sequences, including, notably many members of the C2H2-ZF family which recognize sequences of up to 20 or 30 bp.

To summarize the present study, we developed a method, Signature-QBiC which integrates profiles of the signatures of mutational processes with universal PBM data to predict the likely effects of mutations caused by each of 47 mutational signatures on the binding of each of 582 human TFs. We showed that the GRs and LRs computed from mutational signatures are very similar to GRs and LRs computed from actual somatic promoter mutations observed in tumors dominated by the same mutational signature.

Three interesting generalizations arise from the results in this study. First, mutational signatures that increase or decrease the AT-content of TF binding sites have relatively strong effects on the binding affinity of a broad range of TFs. For most TF classes, signatures dominated by mutations from C to A or T (“+ AT” mutations in Fig. 3b), cause loss of binding, which is consistent with the predominance of cytosines and guanines in the binding sites of most TFs. However, for some TF classes, those with AT-PWMs, + AT mutations in AT-rich sequence tend to decrease sequence entropy and increase binding (Table S8 and Fig. 4a, b). In complementary fashion, for GC-PWM TFs, − AT mutations in GC-rich sequence also tend to decrease entropy and increase binding (Table S9 and Fig. 4c, d). Second, although C>G mutations do not affect AT content, mutational signatures that are dominated by C>G mutations strongly affect TF binding. Surprisingly, this includes homeodomain TFs, even though these recognize AT-rich sequences (e.g. SBS13 in Fig. 3a, b). Third, in contrast to C>G mutations, signatures dominated by T>A mutations have little effect on the binding affinity of TFs. For example, mutational signatures SBS22 and SBS34 have little effect on the binding affinity of any TF class, including homeodomain TFs, even though these recognize AT-rich sequences. Taken together, these generalizations lead to the conclusion that cytosines and guanines are crucial in determining TF-binding and are not interchangeable.

To better understand the possible biological consequences of altered binding affinity by particular signatures, for each signature we investigated the pathways enriched for gain- or loss-of-binding TFs. We found that pathways involved in NOTCH1 signaling and TLR signaling may be affected by mutational processes that are prevalent in some types of cancer. An example affecting NOTCH1 and TLR signaling is UV-induced mutagenesis that causes signature SBS7a. In conclusion, the present study raises the hypothesis that particular mutational signatures may preferentially affect binding of particular classes of TFs in ways that tend to promote particular pathways to oncogenesis.

## Supplementary Information

Supplementary Figures.

Supplementary Tables.
