# Supplementary material for: Mutational processes in cancer preferentially affect binding of particular transcription factors
Source: Sci Rep. 2021 Feb 8;11:3339. doi: 10.1038/s41598-021-82910-0 (PMC7870974; doi:10.1038/s41598-021-82910-0)
Supplement: Supplementary file 1 — Supplementary Figures. [file 41598_2021_82910_MOESM1_ESM.pdf]

Mutational Processes in Cancer Preferentially Affect Binding of Particular Transcription Factors

Mo Liu, Arnoud Boot, Alvin W.T. Ng, Raluca Gordân, Steven G. Rozen

Supplementary Figures

Figure S1 TF binding-domain classes within each TF cluster (A-D). Each color represents a TF binding-domain class.

Figure S2 The proportions of mutations in each mutational signature

The mutations were categorized into “+T” including C>A and C>T, “-T” including T>A and T>C, and “Others” including “C>G” and “T>A”. Each row corresponds to a mutational signature ranked as Figure 3a.

Figure S3 Distribution of GRs and LRs for TFs of each TF binding-domain class.

GRs (red) and LRs (green) for TFs of each TF binding-domain class were plotted as histograms. For each histogram plot, the horizontal axis corresponds to the value GR and LR while the vertical axis corresponds to the density of histogram.

Figure S4 All pathways enriched for affected TFs predicted from Signature-QBiC across 47 mutational signatures.

Each row corresponds to a pathway and each column corresponds to a mutational signature. The category of “Gain”, “Loss” or “Both” indicates enrichment of gain-of-binding TFs, loss-of-binding TFs or both.

Figure S1

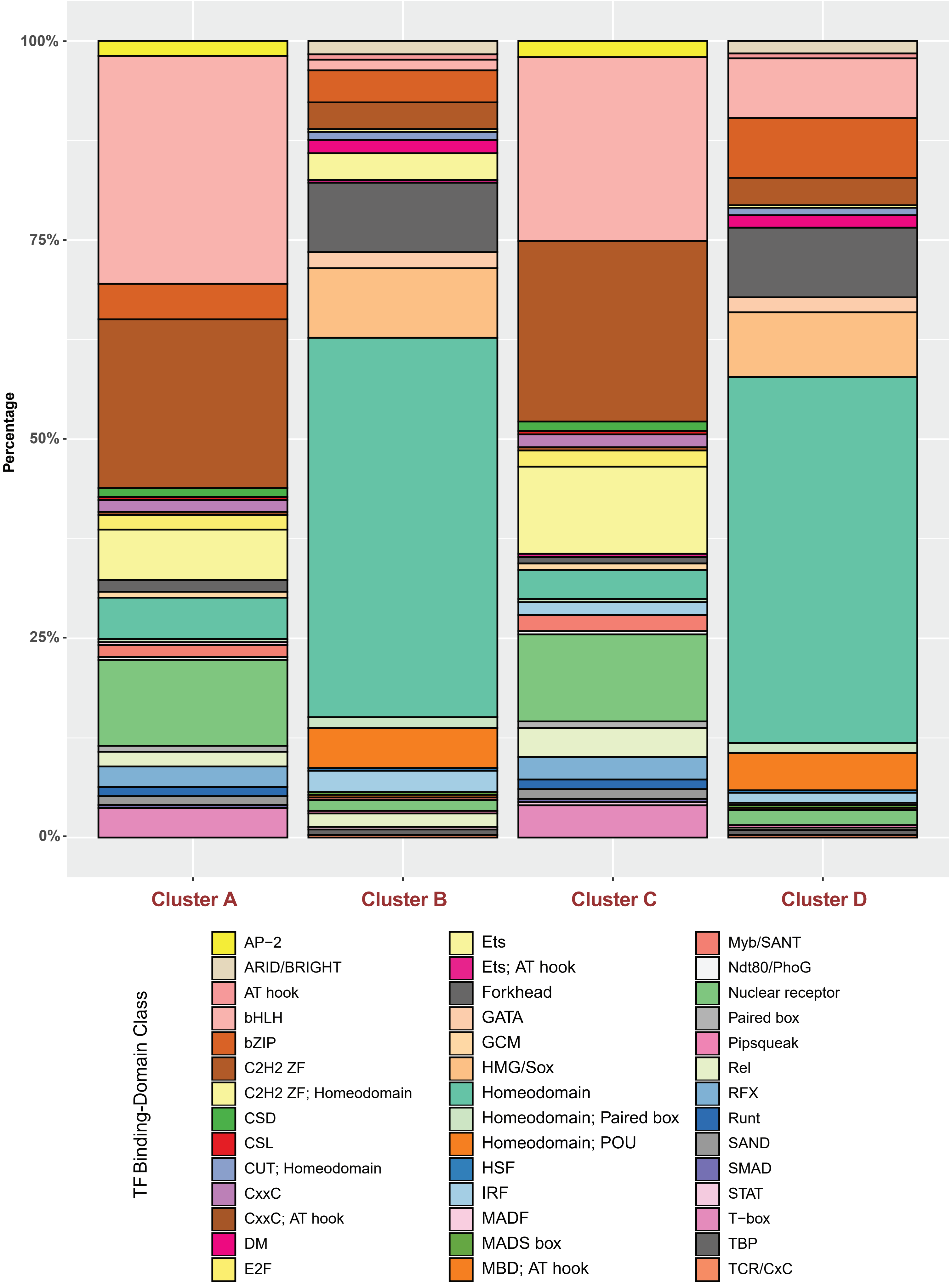
Percentage
100%
75%
50%
25%
0%
Cluster A
Cluster B
Cluster C
Cluster D
TF Binding-Domain Class
AP−2
ARID/BRIGHT
AT hook
bHLH
bZIP
C2H2 ZF
C2H2 ZF; Homeodomain
CSD
CSL
CUT; Homeodomain
CxxC
CxxC; AT hook
DM
E2F
Ets
Ets; AT hook
Forkhead
GATA
GCM
HMG/Sox
Homeodomain
Homeodomain; Paired box
Homeodomain; POU
HSF
IRF
MADF
MADS box
MBD; AT hook
Myb/SANT
Ndt80/PhoG
Nuclear receptor
Paired box
Pipsqueak
Rel
RFX
Runt
SAND
SMAD
STAT
T−box
TBP
TCR/CxC

Figure S2

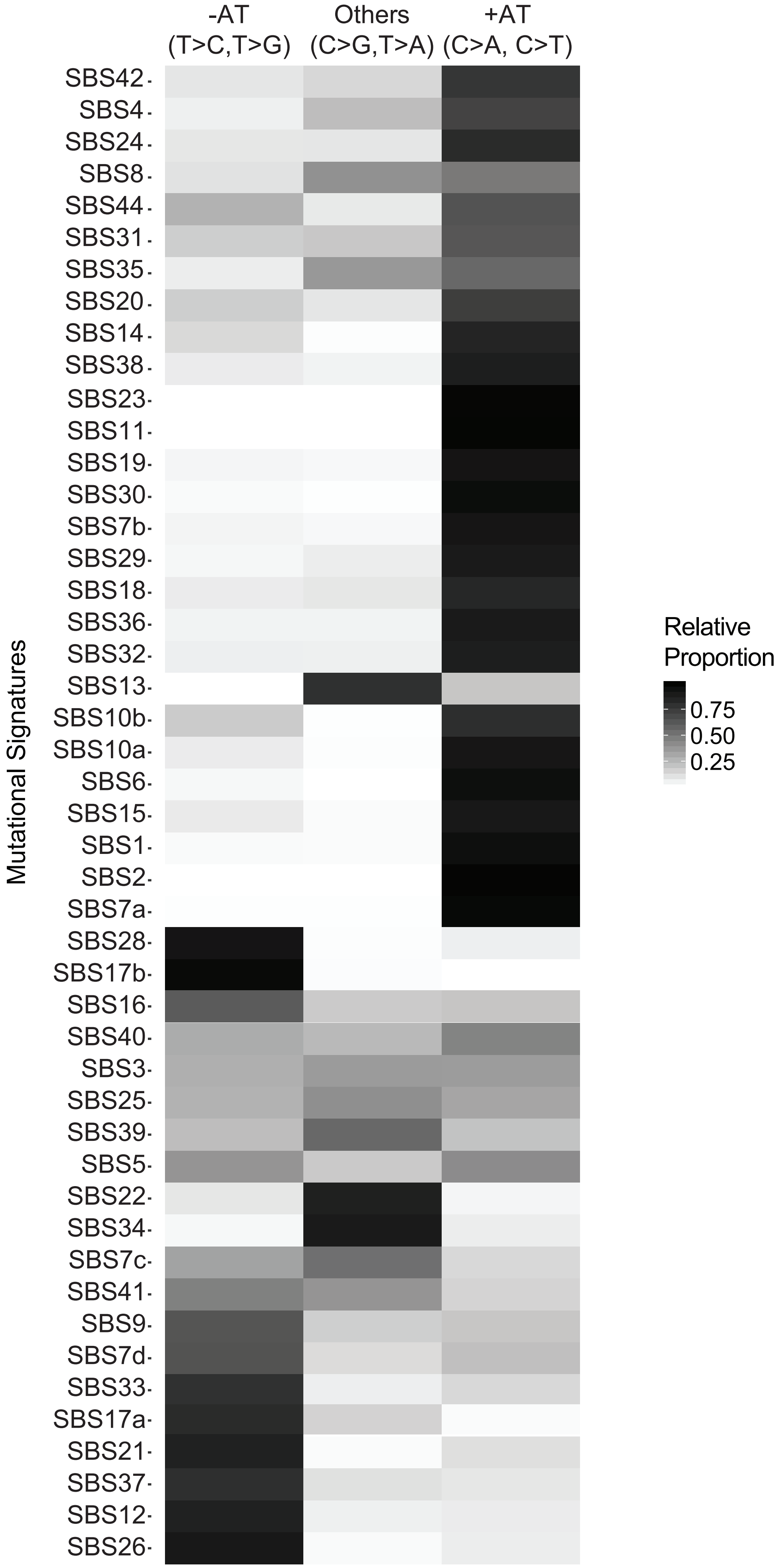
-AT
(T>C,T>G)
Others
(C>G,T>A)
+AT
(C>A, C>T)
Mutational Signatures
SBS42
SBS4
SBS24
SBS8
SBS44
SBS31
SBS35
SBS20
SBS14
SBS38
SBS23
SBS11
SBS19
SBS30
SBS7b
SBS29
SBS18
SBS36
SBS32
SBS13
SBS10b
SBS10a
SBS6
SBS15
SBS1
SBS2
SBS7a
SBS28
SBS17b
SBS16
SBS40
SBS3
SBS25
SBS39
SBS5
SBS22
SBS34
SBS7c
SBS41
SBS9
SBS7d
SBS33
SBS17a
SBS21
SBS37
SBS12
SBS26
Relative
Proportion
0.75
0.50
0.25

Figure S3

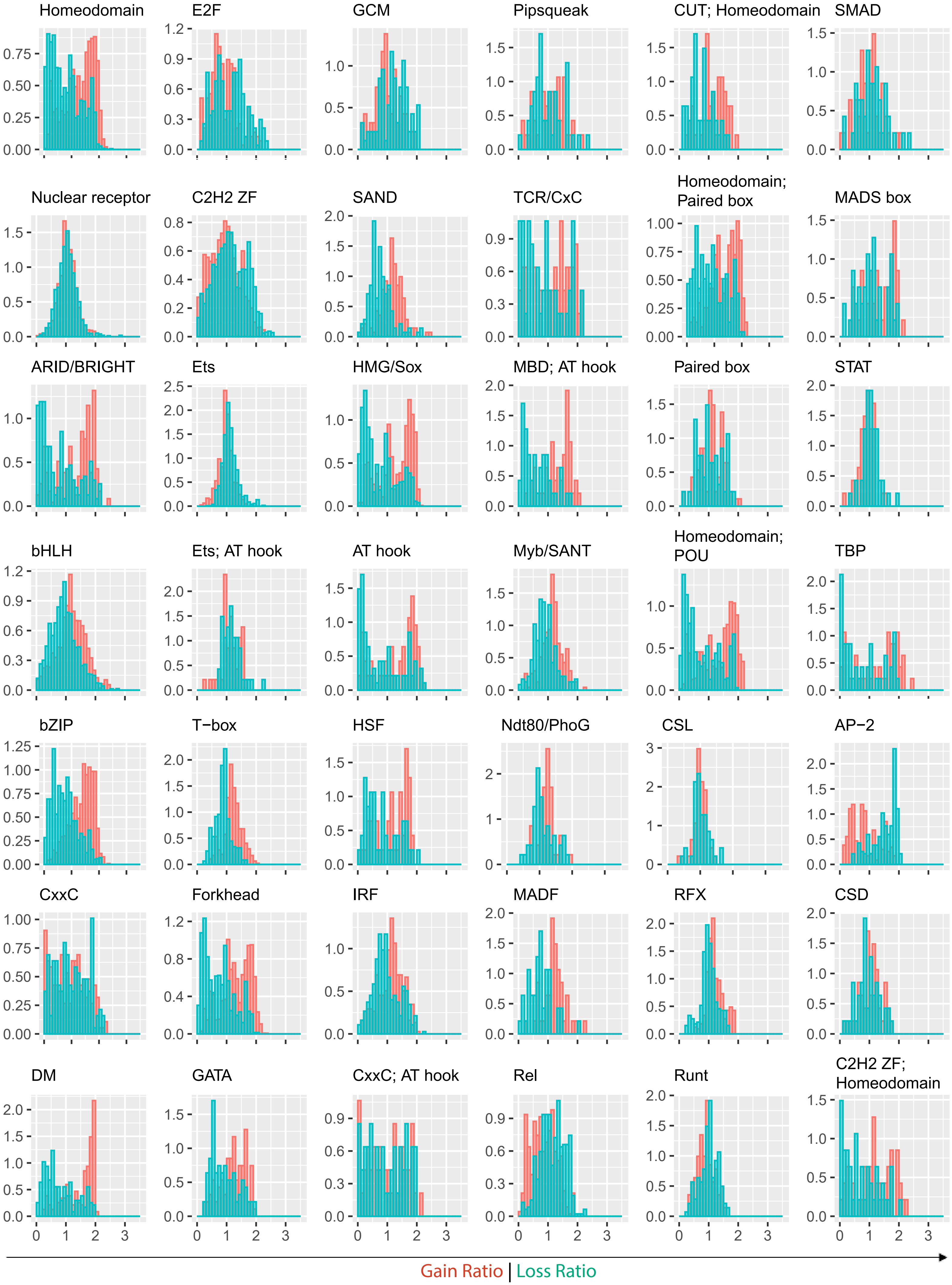
Homeodomain
E2F
GCM
Pipsqueak
CUT; Homeodomain
SMAD
Nuclear receptor
C2H2 ZF
SAND
TCR/CxC
Homeodomain; Paired box
MADS box
ARID/BRIGHT
Ets
HMG/Sox
MBD; AT hook
Paired box
STAT
bHLH
Ets; AT hook
AT hook
Myb/SANT
Homeodomain; POU
TBP
bZIP
T−box
HSF
Ndt80/PhoG
CSL
AP−2
CxxC
Forkhead
IRF
MADF
RFX
CSD
DM
GATA
CxxC; AT hook
Rel
Runt
C2H2 ZF; Homeodomain
0 1 2 3
Gain Ratio | Loss Ratio

Figure S4-1

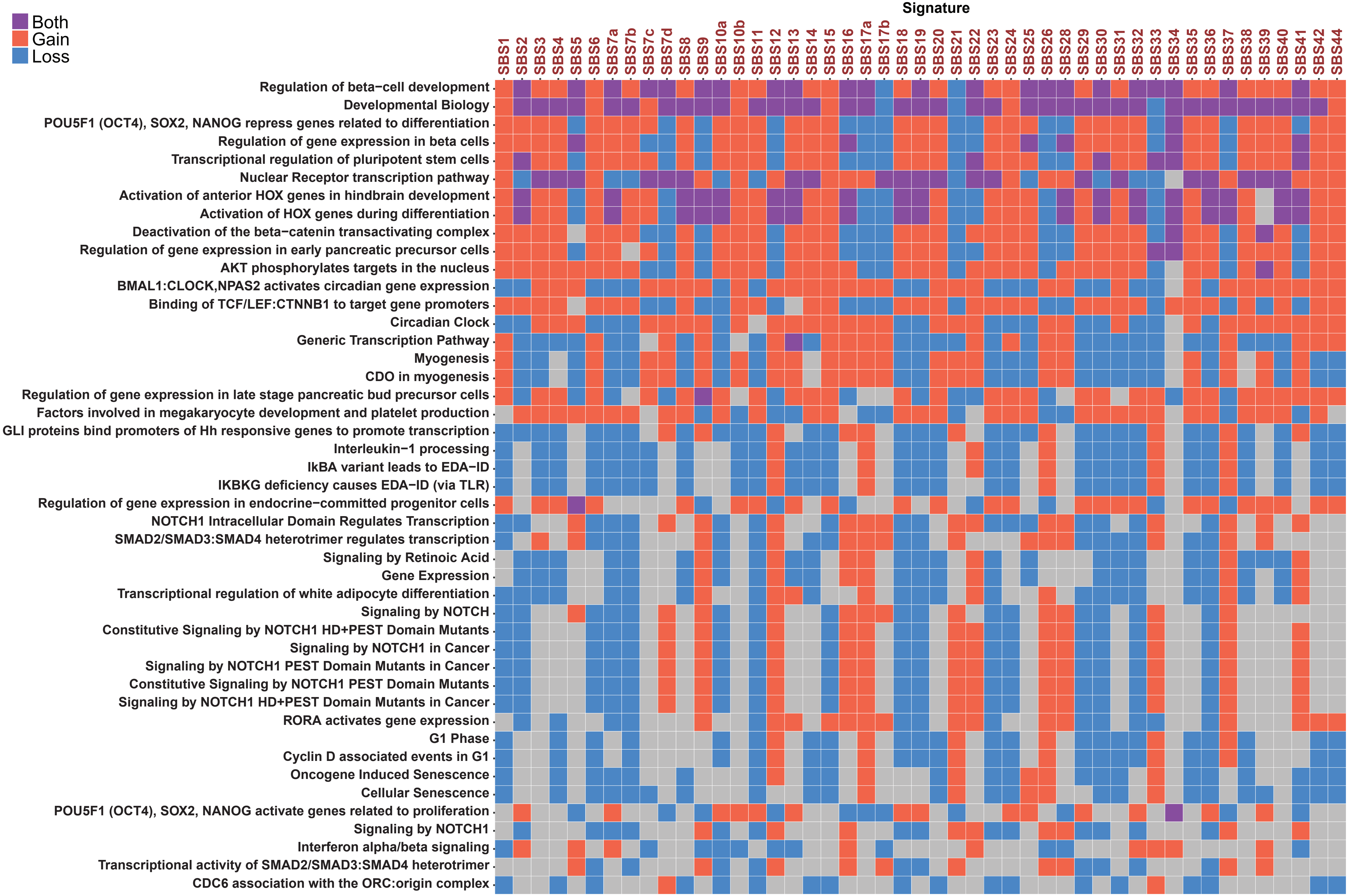
Both
Gain
Loss
Signature
SBS1
SBS2
SBS3
SBS4
SBS5
SBS6
SBS7a
SBS7b
SBS7c
SBS7d
SBS8
SBS9
SBS10a
SBS10b
SBS11
SBS12
SBS13
SBS14
SBS15
SBS16
SBS17a
SBS17b
SBS18
SBS19
SBS20
SBS21
SBS22
SBS23
SBS24
SBS25
SBS26
SBS28
SBS29
SBS30
SBS31
SBS32
SBS33
SBS34
SBS35
SBS36
SBS37
SBS38
SBS39
SBS40
SBS41
SBS42
SBS44
Regulation of beta−cell development
Developmental Biology
POU5F1 (OCT4), SOX2, NANOG repress genes related to differentiation
Regulation of gene expression in beta cells
Transcriptional regulation of pluripotent stem cells
Nuclear Receptor transcription pathway
Activation of anterior HOX genes in hindbrain development
Activation of HOX genes during differentiation
Deactivation of the beta−catenin transactivating complex
Regulation of gene expression in early pancreatic precursor cells
AKT phosphorylates targets in the nucleus
BMAL1:CLOCK,NPAS2 activates circadian gene expression
Binding of TCF/LEF:CTNNB1 to target gene promoters
Circadian Clock
Generic Transcription Pathway
Myogenesis
CDO in myogenesis
Regulation of gene expression in late stage pancreatic bud precursor cells
Factors involved in megakaryocyte development and platelet production
GLI proteins bind promoters of Hh responsive genes to promote transcription
Interleukin−1 processing
IkBA variant leads to EDA−ID
IKBKG deficiency causes EDA−ID (via TLR)
Regulation of gene expression in endocrine−committed progenitor cells
NOTCH1 Intracellular Domain Regulates Transcription
SMAD2/SMAD3:SMAD4 heterotrimer regulates transcription
Signaling by Retinoic Acid
Gene Expression
Transcriptional regulation of white adipocyte differentiation
Signaling by NOTCH
Constitutive Signaling by NOTCH1 HD+PEST Domain Mutants
Signaling by NOTCH1 in Cancer
Signaling by NOTCH1 PEST Domain Mutants in Cancer
Constitutive Signaling by NOTCH1 PEST Domain Mutants
Signaling by NOTCH1 HD+PEST Domain Mutants in Cancer
RORA activates gene expression
G1 Phase
Cyclin D associated events in G1
Oncogene Induced Senescence
Cellular Senescence
POU5F1 (OCT4), SOX2, NANOG activate genes related to proliferation
Signaling by NOTCH1
Interferon alpha/beta signaling
Transcriptional activity of SMAD2/SMAD3:SMAD4 heterotrimer
CDC6 association with the ORC:origin complex

Figure S4-2

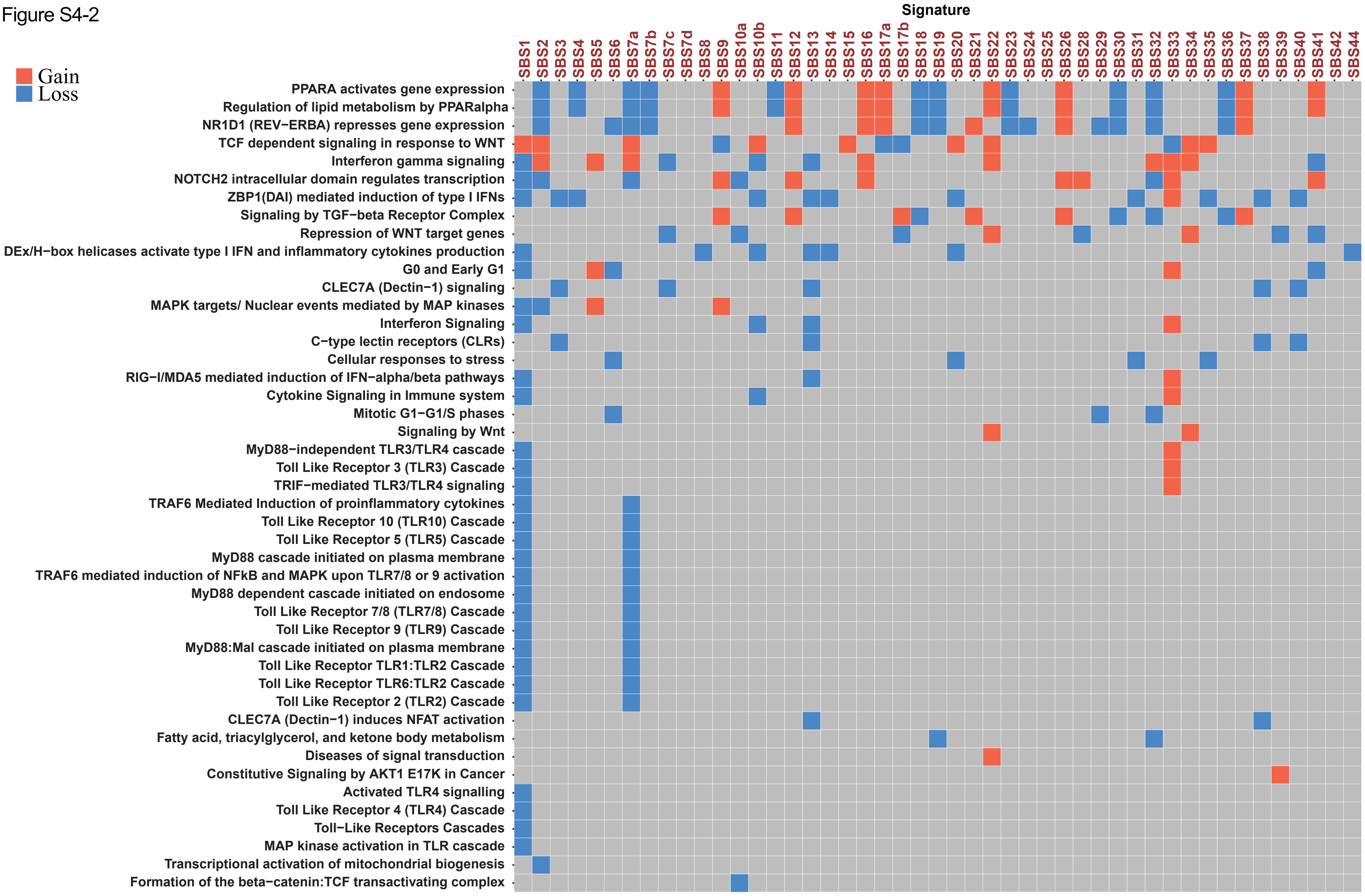
Signature
SBS1
SBS2
SBS3
SBS4
SBS5
SBS6
SBS7a
SBS7b
SBS7c
SBS7d
SBS8
SBS9
SBS10a
SBS10b
SBS11
SBS12
SBS13
SBS14
SBS15
SBS16
SBS17a
SBS17b
SBS18
SBS19
SBS20
SBS21
SBS22
SBS23
SBS24
SBS25
SBS26
SBS28
SBS29
SBS30
SBS31
SBS32
SBS33
SBS34
SBS35
SBS36
SBS37
SBS38
SBS39
SBS40
SBS41
SBS42
SBS44
Gain
Loss
PPARA activates gene expression
Regulation of lipid metabolism by PPARalpha
NR1D1 (REV−ERBA) represses gene expression
TCF dependent signaling in response to WNT
Interferon gamma signaling
NOTCH2 intracellular domain regulates transcription
ZBP1(DAI) mediated induction of type I IFNs
Signaling by TGF−beta Receptor Complex
Repression of WNT target genes
DEx/H−box helicases activate type I IFN and inflammatory cytokines production
G0 and Early G1
CLEC7A (Dectin−1) signaling
MAPK targets/ Nuclear events mediated by MAP kinases
Interferon Signaling
C−type lectin receptors (CLRs)
Cellular responses to stress
RIG−I/MDA5 mediated induction of IFN−alpha/beta pathways
Cytokine Signaling in Immune system
Mitotic G1−G1/S phases
Signaling by Wnt
MyD88−independent TLR3/TLR4 cascade
Toll Like Receptor 3 (TLR3) Cascade
TRIF−mediated TLR3/TLR4 signaling
TRAF6 Mediated Induction of proinflammatory cytokines
Toll Like Receptor 10 (TLR10) Cascade
Toll Like Receptor 5 (TLR5) Cascade
MyD88 cascade initiated on plasma membrane
TRAF6 mediated induction of NFkB and MAPK upon TLR7/8 or 9 activation
MyD88 dependent cascade initiated on endosome
Toll Like Receptor 7/8 (TLR7/8) Cascade
Toll Like Receptor 9 (TLR9) Cascade
MyD88:Mal cascade initiated on plasma membrane
Toll Like Receptor TLR1:TLR2 Cascade
Toll Like Receptor TLR6:TLR2 Cascade
Toll Like Receptor 2 (TLR2) Cascade
CLEC7A (Dectin−1) induces NFAT activation
Fatty acid, triacylglycerol, and ketone body metabolism
Diseases of signal transduction
Constitutive Signaling by AKT1 E17K in Cancer
Activated TLR4 signalling
Toll Like Receptor 4 (TLR4) Cascade
Toll−Like Receptors Cascades
MAP kinase activation in TLR cascade
Transcriptional activation of mitochondrial biogenesis
Formation of the beta−catenin:TCF transactivating complex
